# MUTUAL: Towards Holistic Sensing and Inference in the Operating Room

**DOI:** 10.1007/978-3-031-77610-6_17

**Published:** 2025-01-17

**Authors:** Julien Quarez, Yang Li, Hassna Irzan, Matthew Elliot, Oscar MacCormac, James Knigth, Martin Huber, Toktam Mahmoodi, Prokar Dasgupta, Sebastien Ourselin, Nicholas Raison, Jonathan Shapey, Alejandro Granados

**Affiliations:** 1 Surgical & Interventional Engineering, King’s College London, UK; 2 Neurosurgery Department, King’s College London, London, UK; 3 Department of Urology, Guy’s Hospital, London, UK; 4 Department of Engineering, King’s College London, UK

**Keywords:** Multimodal data, Embodied AI, ROS 2, Surgical data

## Abstract

Embodied AI (E-AI) in the form of intelligent surgical robotics and other agents is calling for data platforms to facilitate its development and deployment. In this work, we present a cross-platform multimodal data recording and streaming software, MUTUAL, successfully deployed on two clinical studies, along with its ROS 2 distributed adaptation, MUTUAL-ROS 2. We describe and compare the two implementations of MUTUAL through their recording performance under different settings. MUTUAL offers robust recording performance at target configurations for multiple modalities, including video, audio, and live expert commentary. While this recording performance is not matched by MUTUAL-ROS 2, we demonstrate its advantages related to real-time streaming capabilities for AI inference and more horizontal scalability, key aspects for E-AI systems in the operating room. Our findings demonstrate that the baseline MUTUAL is well-suited for data curation and offline analysis, whereas MUTUAL-ROS 2, should match the recording reliability of the baseline system under a fully distributed manner where modalities are handled independently by edge computing devices. These insights are critical for advancing the integration of E-AI in surgical practice, ensuring that data infrastructure can support both robust recording and real-time processing needs.

## 1 Introduction

While Artificial Intelligence (AI) has traditionally operated within the digital realm, only recently have new methodologies been proposed to enable agents to interact within a physical environment. Embodied AI (E-AI), supported by the integration of sensors and actuators, allows digital systems to perceive, manipulate, and learn from the physical world [26,32]. Learning through interactions within an environment, known as embodied cognition [25,32], has garnered increasing interest over the years. This interest has shifted the field from conventional AI, which primarily relies on offline datasets and static environments [3], to artificial general intelligence, where AI can adapt to and overcome environmental changes [5]. E-AI holds significant promise for automating tasks prone to human error [5] and adapting to unforeseen events.

Surgery has seen early forms of E-AI through Robotic-Assisted Surgery (RAS) [17,20,30], meeting three out of the four paradigms of E-AI [25]: perception, action, and memory, while lacking continuous learning. RAS has been used to automate tasks of varying complexity, from simple gestures such as knot-tying [29] to entire procedures like knee arthroplasty [14]. Despite these advancements, we argue that surgeons will remain the core agents in the operating room (OR), with E-AI systems enhancing the precision of their technical skills and supporting their situational awareness and decision-making abilities (non-technical skills) to ensure a holistic approach to surgical practice. However, substantial integration of E-AI in the OR is still limited due to the lack of tools necessary to capture the environmental characteristics of the OR [27].

Capturing a holistic scene of the OR is a challenging endeavor [18]. The OR is a highly complex and dynamic environment, and introducing additional recording and streaming technologies can be disruptive [28]. Furthermore, domain expert knowledge is seldom captured during interventions in the OR and is typically limited to manual annotations provided retrospectively, which leads to the loss of valuable information. Moreover, while there has been a push to diversify the modalities captured in the OR, video and imaging remain the primary data sources for AI applications. A multimodal approach to data is expected to increase the understanding of an environment by processing different sources of information similarly to human cognition.

To enable the meaningful development of E-AI, we propose a distributed **mu**l**t**imodal s**u**rgical d**a**ta p**l**atform, MUTUAL, for sensing and inference in the OR. Our contributions are as follows: **1)** two multimodal platforms for efficient recording of data and distributed inference, **2)** integration of real-time expert knowledge and annotations, and **3)** comparative evaluation of both platforms and their demonstration on two clinical scenarios. Code and tutorial can be found on https://github.com/JK-rez/MUTUAL.git.

## 2 Related Work

Few platforms have been deployed and trialled in the OR to capture multimodal intraoperative surgical data, including the OR Black Box^®^, which continuously captures and synchronises several sources of intraoperative data [9], and the Multi-sensing AI Environment for Surgical Task and Role Optimisation (MAESTRO) [24]. The OR Black Box^®^ includes panoramic cameras, microphones, and anaesthesia monitors. In contrast, the MAESTRO platform integrates a wider array of modalities, such as depth cameras, laparoscopic cameras, eye trackers, and several wearable physiological sensors and functional neuroimaging sensors.

These platforms support various downstream tasks, including measuring surgeon cognitive workload [8], automatic surgical check-listing [24], resource optimisation, and education [7]. Despite their advancements, these platforms face significant challenges in the context of E-AI. As surgical data recording becomes increasingly common, the variety and volume of recordings will escalate, necessitating robust connectivity, high Input/Output bandwidth, and abundant storage space for recording platforms. Additionally, if storage devices are not closely integrated with high-performance computing devices, transferring data for training E-AI becomes inefficient. The MAESTRO platform employs the Lab Streaming Layer (LSL) [1] for real-time data streaming. However, LSL is standalone software that only natively supports a limited set of recording devices and lacks integration with common E-AI systems, including robotic systems. Moreover, no multimodal surgical data platform has been rigorously assessed for surgical recording and streaming performance, which is a critical gap given the complex requirements of multimodal data handling.

Performance assessment for multimodal data recording and streaming systems is inherently complex. While substantial work exists in other domains, such as smart city Internet of Things, where platforms like FIWARE undergo load testing and scalability assessments [2], similar efforts are absent in the surgical domain. FIWARE’s testing metrics include CPU and RAM usage, highlighting the need for analogous performance metrics in surgical data systems.

Few studies evaluate inference performance and resource consumption of AI models developed for surgical applications [15]. With the AI research community’s focus on faster inference through methods including model optimisation such as pruning [22], hardware acceleration [4,10,19], and framework optimisation [12], there remains a lack of exploration into efficiently deploying AI, particularly E-AI, in the OR. Considerable research explores leveraging E-AI in surgical robotic systems [6,13,31] and virtual assistants [11,21]. For integrating a data platform with E-AI, the Robot Operating System 2 (ROS 2) [16] has been widely adopted across various domains. This widespread adoption motivates us to design our platform in ROS 2 to address the integration challenges and enhance the capabilities of surgical data platforms for E-AI applications.

In summary, a multimodal surgical data platform that supports a wide range of customisable devices, is performance-tested, and is distributed to enable close integration with E-AI is needed.

## 3 Methods

We propose MUTUAL (Multimodal Surgical Data Platform), a multi-process cross-platform software utilising open-source Software Development Kits (SDKs), FFmpeg and Shell scripts to capture multimodal surgical data. We also introduce MUTUAL-ROS 2 which extends MUTUAL for more native support for E-AI applications using ROS 2. A Graphical User Interface (GUI) is designed to facilitate the acquisition of expert knowledge alongside the data captured by these two platforms.

### Data Modalities and Equipment

We demonstrate the performance of our proposed platform across multiple and diverse modalities (Table 1) captured simultaneously. These modalities are illustrative inputs that an agent would potentially need access to capture the knowledge of the environment at a given time. These modalities can be connected to laptops or edge computing devices in the OR and/or in a Control Room, a room adjacent to the OR used for monitoring surgical interventions by other clinical team members or by the surgical team during tele-operated interventions.

### 3.1 Multi-modal Platform Overview

#### MUTUAL (baseline)

**Fig. 1:**
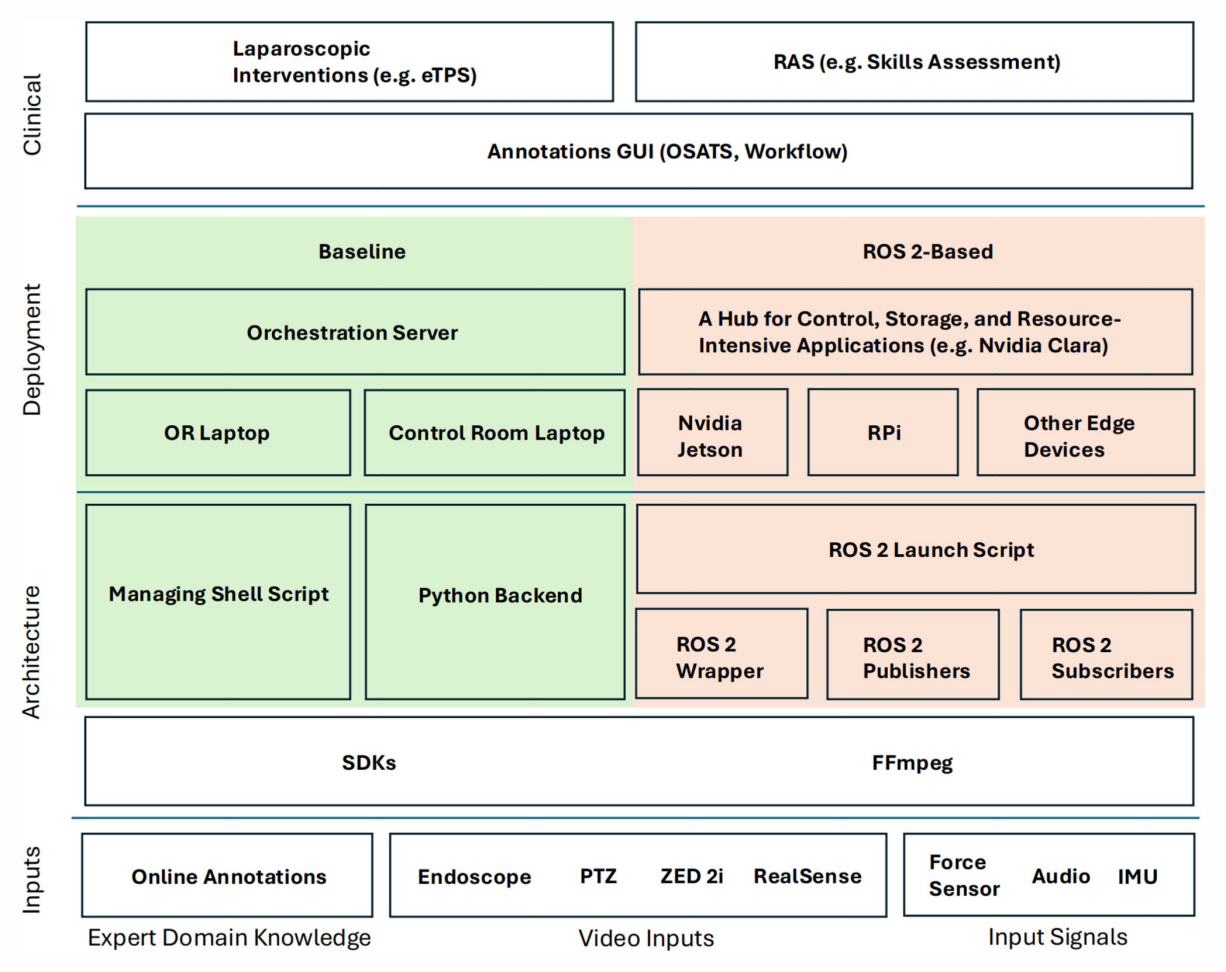
Overview of MUTUAL and MUTUAL-ROS 2.

A Shell script is used to launch and manage the recording (Fig. 1). This script calls each of the device’s SDKs as a separate process while recording their process IDs (PIDs). SDKs were modified to wait in an endless loop once all respective initialisation steps are performed. Every device has varying initialisation times after launch, allowing devices to “wait” for one another, is one way to ensure small differences in synchronisation. The Shell scripts check that all devices are initialised and ready to record before allowing the generation of the starting criterion through a terminal or Secure Shell (SSH) command. Once recording has started, the Shell script monitors device connectivity and error handling using the PIDs. The file sizes are also checked to make sure data is stored throughout. Error-catching protocols are incorporated in case a device fails due to a killed process or due to a file not increasing in size. Ignore the error, restart the concerned process, or intervene on the device are some of the options a user could consider. Recording are also contained in an endless loop, waiting for a stop criterion that will kill all processes and clean temporary files. A Python script is used to launch and manage all FFmpeg recordings in the control room. Once recording is triggered, a Python subprocess call will send a start criterion through an SSH command to the managing Shell script via the orchestration server. This allows for relatively synchronised recording between the two laptops used in the eTPS study. See section 3.2. Once a stop button is clicked on the GUI, Python will kill all FFmpeg processes and send a stop criterion to the managing Shell script through the orchestration server. See supplementary material for overview of recording workflow.

#### Mutual-Ros 2

MUTUAL was adapted to ROS 2 to investigate methods for further minimising start/stop latency, increasing horizontal scalability, adopting a unified storage format (rosbag2), and enabling real-time streaming for deploying E-AI applications. The building blocks of MUTUAL-ROS 2 are illustrated in Fig. 1. Using the publisher and subscriber mechanism to interact with data sources, we can leave the data publishers running and subscribe to them for any applications on any devices connected to the ROS 2 network.

#### Expert Knowledge Acquisition and Input Orchestration GUI

A bottleneck towards the translation of AI models into the OR has been a lack of annotations [18]. Allowing for real-time annotations during a procedure removes much of the process’s friction and facilitates the creation of quality datasets. Through the clinically tailored GUI, clinicians can start and stop recording, annotate workflow, assign skill levels (OSATS scores [23]), and write comments on how the procedure is being performed (see supplementary material).

### 3.2 Experimental Design

MUTUAL has been deployed for two real-world surgical data studies (Fig. 1 top) requiring multimodal data: **1)** a study for endoscopic Transsphenoidal Pituitary Surgery (eTPS) using phantom models in a mock OR at St Thomas’ Hospital, London, UK, and **2)** a study for surgical skills assessment for RAS at Guy’s Hospital, London, UK. The GUI was developed using the open-source version of PySimpleGUI. In our study, MUTUAL consists of two laptops (one in the mock OR and one in the control room interconnected through an orchestration server), whereas MUTUAL-ROS 2 consists of a laptop and an edge computing device. The performance metrics evaluated include CPU and RAM usage, start and stop latency, recording size, as well as sampling frequency. We record data from the first four modalities simultaneously, using the specification outlined in Table 1, running the experiment for ten minutes. The four chosen modalities are the ones deployed in the OR for the eTPS study as they are the most likely to be integrated into an edge computing device. All experiments are carried out with a laptop (CPU: i7-11800H @ 2.30G, RAM: 16 GB DDR4-3200) and a Nvidia Jetson Orin Nano 8GB developer kit to control the number of variables. The ROS 2 network router is the TP-Link BE9300 Tri-Band Router.

We compare the performance of MUTUAL and MUTUAL-ROS 2 platforms against a group of metrics. We monitor only the subscriber nodes that save incoming data into ROS 2 bags in the MUTUAL-ROS 2. The subscriber nodes in MUTUAL-ROS 2 offer the closest functionality to MUTUAL, and therefore, they are the only monitored processes. Additionally, the subscriber nodes on the laptop are initialised with either a local (laptop) or networked (Jetson) publisher to account for potential bandwidth limitations caused by either network conditions or ROS 2 middleware implementation. We refer to the two setups as "Local Sub" and "Networked Sub", respectively.

## 4 Results

**Fig. 2:**
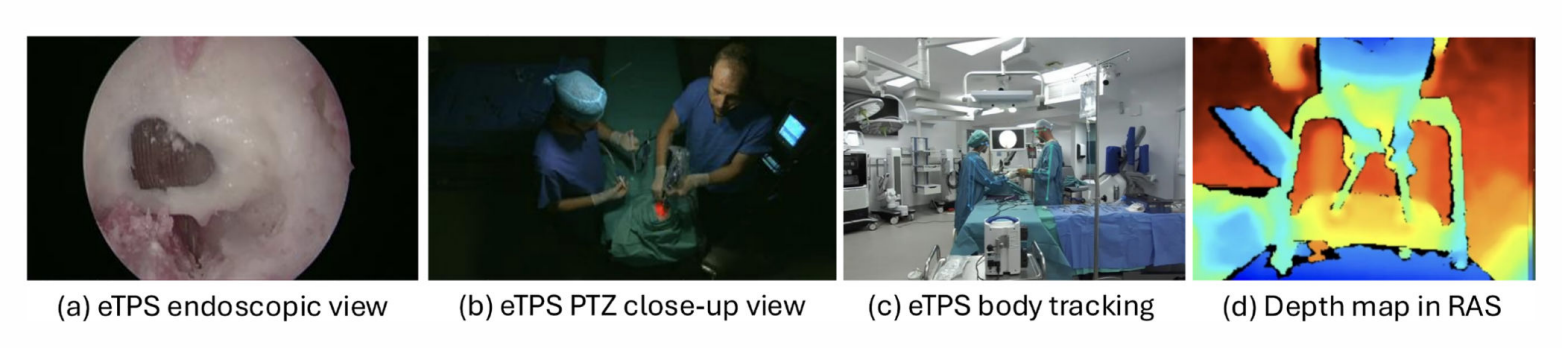
Sample data captured during eTPS and RAS skills assessment studies.

We demonstrate the modalities captured during our experiments during two clinical scenarios, i.e. eTPS and RAS skills assessment in Fig. 2. Under such clinical scenarios, MUTUAL can maintain the configured recording resolution throughout multiple one-hour recording sessions per day. The GUI used during the eTPS study allowed for annotations (workflow and OSATS scores), recording of commentary, and recording orchestration, giving full control to the clinical expert (see Supplemental material).

**Fig. 3:**
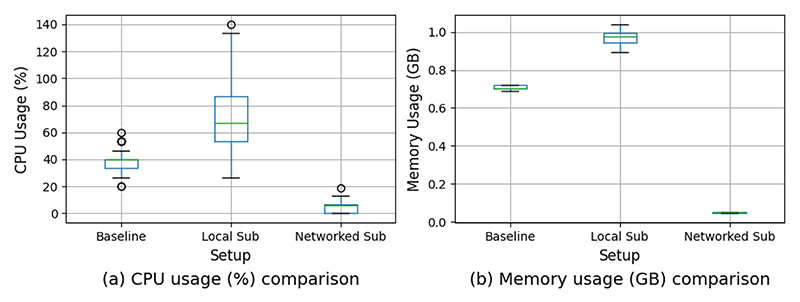
CPU&RAM usage when recording four modalities on a laptop over 10 minutes. CPU usage (%) is expressed in per core capacity. *Baseline*: MUTUAL; *Local Sub*: MUTUAL-ROS 2 where publishers and subscribers are on the same machine; *Networked Sub*: MUTUAL-ROS 2 where publishers and subscribers are on different machines.

In relation to the comparison between MUTUAL and MUTUAL-ROS 2 over ten minutes, the ROS 2 Local Sub setup consumes the most resources (CPU and RAM usage) while the ROS 2 Networked Sub setup consumes the least resources; the difference between their median value is approximately 60% (Fig. 3). With the Networked Sub setup, the frequency of recording drops, especially for video modalities, where the recording frequency is approximately halved (see Table 3). MUTUAL is able to maintain the configured recording frequency over the 10-minute period as expected. Furthermore, we observe that when running the Local Sub setup on the Jetson, the recording frequency increases compared to running the same setup on the laptop. As shown in Table 3, the recording frequency more than tripled for the RealSense device. The decrease in recording frequency with the Networked Sub setup is also reflected in the size of the recording, as illustrated in Table 2. The Local Sub configuration resulted in larger storage consumption even at a lower recording frequency compared to MUTUAL. With the Networked Sub configuration, it also takes more than eight times longer to discover all the topics for subscription and recording. As shown in Table 2, MUTUAL-ROS 2 with Local Sub setup offers the lowest start/stop latency.

## 5 Discussion

Capturing heterogeneous annotated surgical data is critical for developing E-AI methodologies into the OR. Furthermore, ensuring the real-time streaming of this data will facilitate large-scale integration of E-AI. Our proposed platform addresses both of these components effectively. Through our proposed platforms, we showcase how varied modalities can be seamlessly integrated into one to capture a holistic view of the OR. The designed GUI allowed clinical expertise to be captured in real-time, through clinical context via comments, audio recording and OSATS, data which has historically been lacking in the field. Even when AI models will eventually avoid manual annotations, expert knowledge could be used in real-time to validate such predictions.

MUTUAL was deployed successfully in two different environments. During the eTPS study, 24 one-hour data samples were recorded, whereas during the robotic-assisted training, 50 ten-minute samples were recorded. The platform setup took less than an hour in each scenario, and no modifications to the training setup were needed for deployment during RAS training.

MUTUAL baseline’s performance meets the target configuration under the clinical use cases and the experiments. This makes it suitable for recording and dataset curation applications. Still, it lacks the streaming capability or horizontal scalability of MUTUAL-ROS 2, both of which are essential for deploying E-AI applications effectively in the OR. Although the framerate performance of MUTUAL-ROS 2 does not meet the configured recording criteria, it meets the inference rate for many currently AI models running inference on video at 1Hz. Moreover, when running only one modality per device using the Jetson edge computing device, the framerate performance can reach the recording criteria with performance similar to MUTUAL, demonstrating the value of distributed inference. Lastly, MUTUAL does not provide a unified storage format and the same level of start/stop synchronisation achieved by the Local Sub version of MUTUAL-ROS 2.

Despite its competitive performance, our platform can still be further improved. Plug-and-play capabilities are needed for the platform, since a technician is still required to be present for device set up and initialisation. Moreover, the GUI is procedure-specific and requires expertise for adapting it to different clinical scenarios. Last, although the streaming performance of MUTUAL-ROS 2 has not been tested for inference we demonstrate how our platform can support edge computing devices in a distributed manner.

## 6 Conclusion

In this work, we propose two multimodal platforms, MUTUAL and MUTUAL-ROS 2, that are evaluated on two real-world clinical scenarios and compared against resource consumption, start/stop synchronisation, storage, and frequency. Both platforms provide a step towards holistic sensing and inference in the OR, empowering the development and deployment of E-AI, especially robotic E-AI applications. Through careful design choices, the platform allows seamless integration in varied environments and should allow increased data capture at the feature and expert knowledge levels.

## Supplementary Material

Supplementary Figures

## Figures and Tables

**Table 1:** Summary of modalities used in our study. While all modalities are included in the endoscopic study (eTPS), only the first two modalities are included in the RAS skills assessment scenario. When using MUTUAL-ROS 2 data is stored in rosbag files.

Modality	Manufacturer	Resolution @ (Hz)	Storage	Captured Content
ZED 2i	StereoLabs	1080p*2 @15	SVO	RGB-D and body tracking
RealSense	Intel	480p*2 @15	bag	RGB-D and hand pose
Force Sensor	ATI	@ 15	txt	Forces/torque (pantoms)
IMU	MBIENTLAB	@ 10	txt	Hand movement
PTZ	Panasonic	1080p @30	AVI	Overall and hands view
Endoscope	Karl Storz	1080p @30	AVI	Endoscopic video
OR Audio	Sennheise	N/A @48*10^3^	WAV	Conversations
Expert Audio	HyperX	N/A @48*10^3^	WAV	Expert commentary
Annotations	N/A	N/A	txt	OSATS and comments

**Table 2:** Start and stop latency and size of recording comparisons between platforms for the four assessed modalities running simultaneously, measured on the laptop.

Metrics	Baseline	ROS 2 (Local)	ROS 2 (Networked)
Start/Stop Latency (ms)	639	142	1211
Size of Recording (10 min)(GB)	53	97	3.6

**Table 3:** Recording frequencies in Hz.

Modality @ (Hz)	Baseline	Local Sub (Laptop/Jetson)	Networked Sub
ZED Cam Video @15	15	3.2 / 5.5	1.0
RealSense Cam Video @15	15	3.2 / 11.3	1.0
Force Sensor @15	15	14.9 / 15	14.5
IMU @10	10	9.9 / 9.9	9.6
