# Supplementary figures and images for: MUTUAL: Towards Holistic Sensing and Inference in the Operating Room
Source: Med Image Comput Comput Assist Interv. Author manuscript; Available in PMC 2025 Jan 24. (PMC7617325; doi:10.1007/978-3-031-77610-6_17)

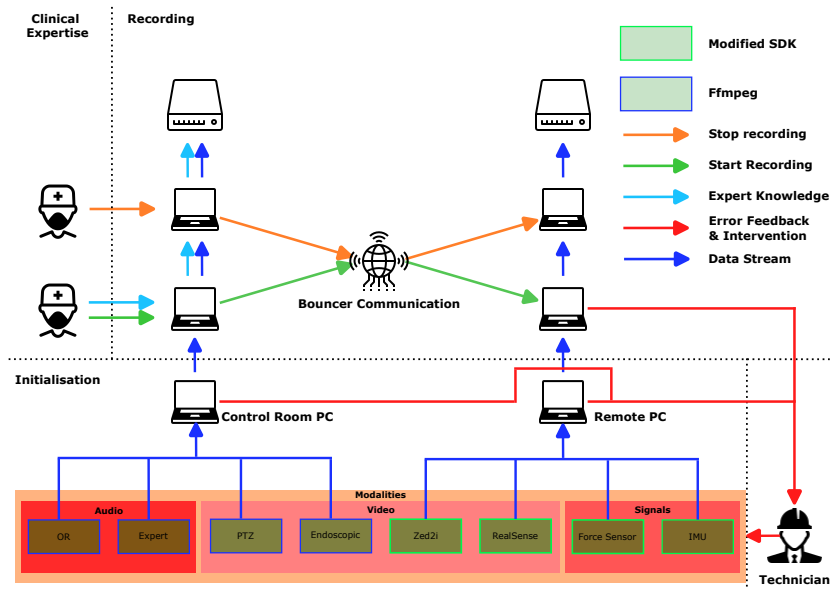

Fig. 1. Overview of recording workflow.

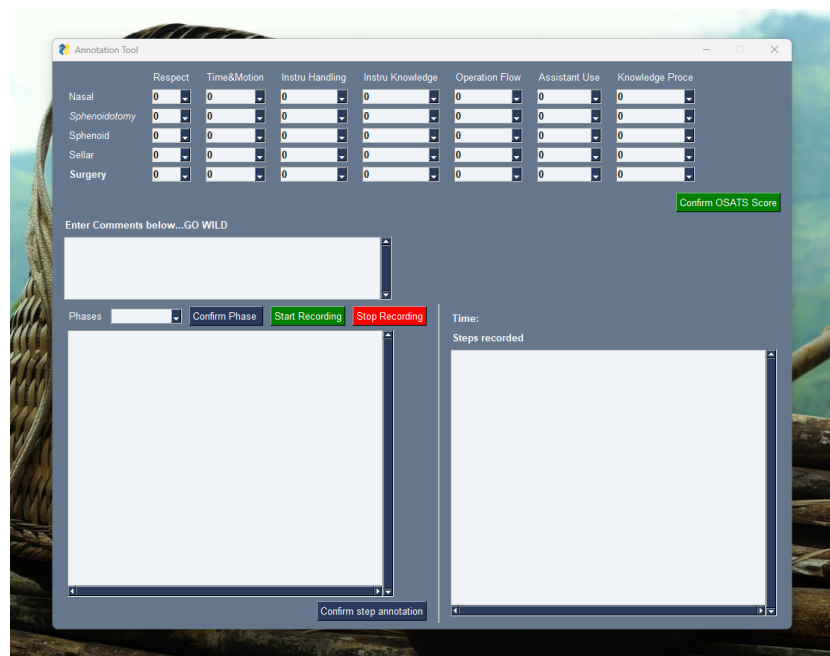

Fig. 2. Clinical GUI.

Supplement: Supplementary Figures [file EMS197965-supplement-Supplementary_Figures.pdf]
